# Photocatalytic H_2_ Production by Visible Light on Cd_0.5_Zn_0.5_S Photocatalysts Modified with Ni(OH)_2_ by Impregnation Method

**DOI:** 10.3390/ijms24129802

**Published:** 2023-06-06

**Authors:** Bence Páll, Maali-Amel Mersel, Péter Pekker, Éva Makó, Veronika Vágvölgyi, Miklós Németh, József Sándor Pap, Lajos Fodor, Ottó Horváth

**Affiliations:** 1 Research Group of Environmental and Inorganic Photochemistry, Center for Natural Sciences, Faculty of Engineering, University of Pannonia, P.O. Box 1158, H-8210 Veszprém, Hungary; pallbence2001@gmail.com (B.P.); sam003miloo@gmail.com (M.-A.M.); fodor.lajos@mk.uni-pannon.hu (L.F.); 2 Environmental Mineralogy Research Group, Research Institute of Biomolecular and Chemical Engineering, University of Pannonia, P.O. Box 1158, H-8210 Veszprém, Hungary; pekkerpeter@gmail.com; 3 Department of Materials Engineering, Research Center for Engineering Sciences, University of Pannonia, P.O. Box 1158, H-8210 Veszprém, Hungary; kristofne.mako.eva@mk.uni-pannon.hu; 4 Research Group of Analytical Chemistry, Center for Natural Sciences, Faculty of Engineering, University of Pannonia, P.O. Box 1158, H-8210 Veszprém, Hungary; vagvolgyi.veronika@mk.uni-pannon.hu; 5 Surface Chemistry and Catalysis Department, Centre for Energy Research, Hungarian Academy of Sciences, Konkoly-Thege Street 29-33, H-1121 Budapest, Hungary; nemeth.miklos@ek-cer.hu (M.N.); pap.jozsef@ek-cer.hu (J.S.P.)

**Keywords:** photocatalysis, hydrogen production, CdS, ZnS, Ni-modification, impregnation

## Abstract

Nowadays, the study of environmentally friendly ways of producing hydrogen as a green energy source is an increasingly important challenge. One of these potential processes is the heterogeneous photocatalytic splitting of water or other hydrogen sources such as H_2_S or its alkaline solution. The most common catalysts used for H_2_ production from Na_2_S solution are the CdS-ZnS type catalysts, whose efficiency can be further enhanced by Ni-modification. In this work, the surface of Cd_0.5_Zn_0.5_S composite was modified with Ni(II) compound for photocatalytic H_2_ generation. Besides two conventional methods, impregnation was also applied, which is a simple but unconventional modification technique for the CdS-type catalysts. Among the catalysts modified with 1% Ni(II), the impregnation method resulted in the highest activity, for which a quantum efficiency of 15.8% was achieved by using a 415 nm LED and Na_2_S-Na_2_SO_3_ sacrificial solution. This corresponded to an outstanding rate of 170 mmol H_2_/h/g under the given experimental conditions. The catalysts were characterized by DRS, XRD, TEM, STEM-EDS, and XPS analyses, which confirmed that Ni(II) is mainly present as Ni(OH)_2_ on the surface of the CdS-ZnS composite. The observations from the illumination experiments indicated that Ni(OH)_2_ was oxidized during the reaction, and that it therefore played a hole-trapping role.

## 1. Introduction

One of the major challenges of the 21st century is the use of renewable energy sources that do not emit CO_2_ during either production or consumption. Hydrogen can be such a material if it is produced by renewable energy sources such as solar energy. This has prompted several research groups to focus in more detail on heterogeneous photocatalytic hydrogen generation [1,2,3]. Although the simplest and most environmentally friendly method for this would be the production of H_2_ by water splitting [2,4,5], the quantum efficiency of this process is relatively low for industrial applications. Much greater efficiency can be achieved by splitting H_2_S or its alkaline solution [6]. H_2_S is a toxic by-product of petroleum refining and natural gas purification, and therefore its photocatalytic splitting into H_2_ and sulphur is also environmentally significant. The most efficient photocatalysts for this process are sulfide-type catalysts [7], mainly those containing CdS [2,3,8,9,10,11]. However, since the conduction band potential of CdS is not sufficiently negative for the reduction of water in a strongly alkaline medium, such as Na_2_S solution, it is often modified with other compounds, especially with ZnS [12,13,14,15,16,17] or MnS [18,19,20,21,22]. He and Guo, however, achieved an outstandingly high H_2_ generation efficiency of 74.6% by co-precipitation of CdS and 5% NiS [23]. Although only few researchers have investigated the optimal preparation conditions of these catalysts under similar irradiation circumstances [24] in detail, several authors have concluded that the 1:1 molar ratio of CdS-ZnS catalysts is the most active [15,25]. It was reported in several publications that hydrothermal treatment enhances the hydrogen production activity of CdS-ZnS composites [16,25,26,27,28,29]. This effect was mainly attributed to the formation of sphalerite-wurtzite twin boundaries during hydrothermal treatment, which causes a lower probability of recombination of photogenerated charge carriers [24,28,29,30,31,32].

A widely studied method to increase the efficiency of a catalyst is to use a co-catalyst. Among these, Ni-containing ones have also proven to be the most efficient, such as metallic Ni [18,33], NiS [23,25,27,34,35,36,37], NiO [38], Ni(OH)_2_ [39,40,41,42], or Ni_2_P [26,43]. In some cases, the Ni-modification was performed by co-precipitation of CdS and ZnS [23,44], but in most cases NiS or Ni(OH)_2_ was subsequently precipitated onto the surface of the CdS-ZnS composite by Na_2_S [27], thioacetamide [23], thiourea [35,45], or NaOH [41,42]. The role of the Ni compound was mainly interpreted as an agent that traps the CB electrons [27,35]. Many studies have previously reported that Ni(OH)_2_ as a co-catalyst contributed to the enhancement of the photocatalytic hydrogen production due to the interface between Ni(OH)_2_ and ZnS-CdS catalyst and/or the photoreduction of nickel(II) to its metallic form, which can improve the charge separation in/on the catalyst [39,40,41,42]. He and Guo attributed the efficiency-enhancing role of NiS to the fact that NiS forms a p-n junction with CdS, resulting in electron transfer from the conduction band of NiS to that of CdS. In addition, the role of the stacking fault structure has been highlighted [23]. In the present work, we apply a non-conventional, impregnation method to modify the Cd_0.5_Zn_0.5_S catalyst with Ni(II), which produces an efficient photocatalyst without costly thermal treatment. Based on our results, we propose a new, alternative mechanism for the operation of the Ni-containing co-catalyst.

## 2. Results and Discussion

Since our long-term goal is to produce the most efficient photocatalyst suitable for developing hydrogen from Na_2_S-containing solution under visible light, our studies have been primarily focused on photocatalytic experiments. Hence, the photocatalytic activity of each of the catalysts prepared was investigated, and thus these results are presented first.

### 2.1. Photocatalytic Hydrogen Production

Three different methods were used to prepare Ni(II)-modified Cd_0.5_Zn_0.5_S catalysts. Two of them are used by several research groups. The most common is the surface modification, in which both Ni(II) and a sulfide precursor are added to the previously prepared Cd_0.5_Zn_0.5_S catalyst to precipitate NiS. In the present work, this composite is designated CZS-10Ni-S. In another, less frequently used procedure, sulfide species are precipitated together from a solution containing both Cd(II), Zn(II), and Ni(II) salts. The resulting “bulk-modified” composite is designated CZS-10Ni-B. In the third technique (impregnation method, labeled “I”), which is investigated in more detail in the present work, the appropriate amount of Ni(II) precursor is added to the aqueous suspension of the previously prepared Cd_0.5_Zn_0.5_S catalyst and allowed to stand overnight. During this time, some of the Ni(II) ions may either be bound into the CdS-ZnS crystal lattice, replacing Cd(II) or Zn(II) ions, or adsorbed on the surface of the particles. The non-bonded Ni(II) precursor was removed by centrifugation and a single wash, and only afterwards Na_2_S was added. The non-bonded Ni(II) and the released Cd(II) and Zn(II) were measured by ICP-OES analysis. In the case of the CZS-10Ni-I sample, only 45% of Ni was bound on the surface before deposition with Na_2_S, while the amount of Cd and Zn released was 1.0% and 2.0%, respectively, compared to the amount of Ni initially added.

The labels of the catalysts prepared are summarized in Table 1. Details of the preparative procedure are described in detail in the experimental section.

**Figure 1:**
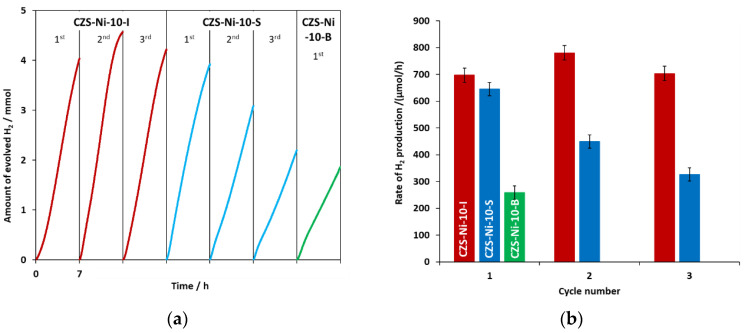
The amount of hydrogen evolved in time (**a**) and the average RHPs calculated from it (**b**). The red, blue, and green colors represent the catalysts CZS-10-I, CZS-10-S, and CZS-10-B, respectively, in both (**a**,**b**). For catalysts CZS-10-I and CZS-10-S, the results for the three consecutive illuminations are presented. The 30 mL of irradiated suspension contained 0.145 M Na_2_S, 0.19 M Na_2_SO_3_, and 20 mg of catalyst in each case.

The results of photocatalytic hydrogen evolution reactions with catalysts modified by the addition of 1% Ni(II), using different procedures under the same conditions, are shown in Figure 1 and Figure S1. During the first illuminations of the catalysts, the composite prepared by impregnation showed the highest rate of hydrogen production (RHP) (697 μmol/h), followed by CZS-10Ni-S (645 μmol/h) with an efficiency of about 10% lower. However, the bulk-modified sample had an efficiency of only about one third of that of the impregnated catalyst (259 μmol/h). The stability of the two best catalysts was also investigated. For this purpose, the catalysts recovered by centrifugation after the first illumination were illuminated twice more with a sacrificial solution of the same composition and concentrations of the constituents as the first one. While the efficiency of CZS-10Ni-S was reduced by half, the one of CZS-10Ni-I even increased slightly. During the second illumination with the visible light LED, the highest activity was measured (781 μmol/h), which is one of the highest values (39.0 mmol/g/h) relative to catalyst mass (see Video S1 in the Supplementary Materials (SM)).

**Figure 2:**
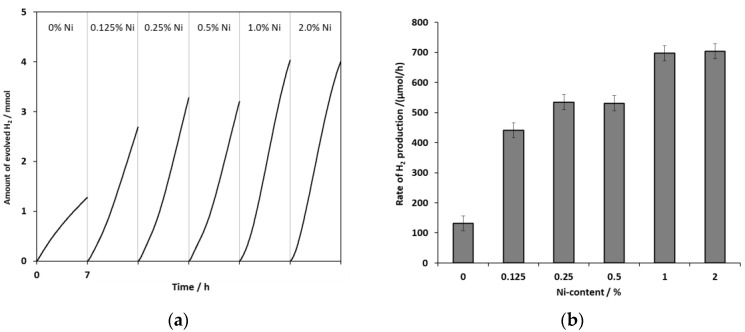
The amount of hydrogen evolved in time (**a**) and the average RHPs (**b**) obtained for catalysts modified by impregnation method with different amounts of Ni(II) (CZS-xxNi-I).

For the impregnation method, which was found to be the best, the influence of Ni content was also investigated. The activity of catalysts prepared with an addition of 0–2% Ni(II) was compared in Figure 2 and Figure S2a. Already at low Ni content, the measured RHP increased more than threefold compared to the unmodified CZS. A Ni content of more than 1% did not increase the efficiency beyond the measurement error.

**Figure 3:**
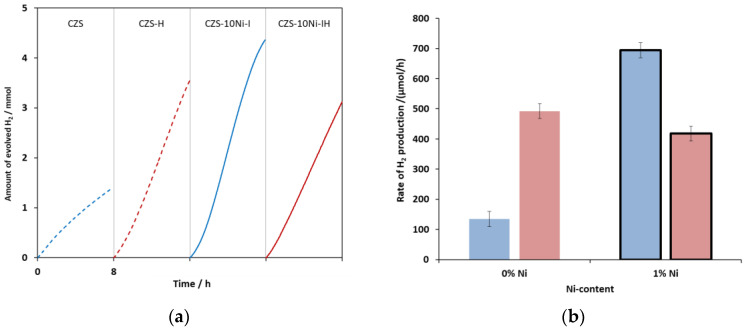
Comparison of the hydrogen evolved over time (**a**) and RHPs (**b**) of hydrothermally treated (red) and non-treated (blue) catalysts unmodified (dashed lines and non-bordered bars) and modified with 1% of Ni(II) by impregnation (solid lines and bordered bars).

Since many research groups use hydrothermal treatment and we have also previously reported that hydrothermal treatment increased the efficiency of CdS-ZnS type catalysts due to the formation of twin boundaries, we investigated the effect of heat treatment on the CZS-10Ni-I composite. As expected, the measured RHP for unmodified CZS increased by more than 3.5 times, meanwhile a decrease of 40% was measured for the Ni-modified catalyst (Figure 3 and Figure S3). During the hydrothermal treatment, the grains were aged due to Ostwald ripening [46], leading to larger particle sizes. Moreover, several research groups have pointed out that the heat treatment promotes the formation of sphalerite-wurtzite twin boundaries, which significantly reduces the probability of e^−^-h^+^ recombination [28,31,47]. This is the main reason for the increase in the activity of the CZS catalyst. However, after Ni-modification, the hydrothermal treatment reduced the efficiency. One possible reason for this is that the increasing particle size leads to a smaller specific surface, so that Ni(OH)_2_ covers the surface in a higher fraction, thus reducing the ratio of photons absorbed by the CdS-ZnS catalyst. Unusually, we investigated not only the initial stage of H_2_ evolution, but also the complete reaction (i.e., the illumination was continued until the sulfide was completely used up). Several research groups have used concentrations of 0.35 M Na_2_S and 0.25 M Na_2_SO_3_ for a better quantum yield (Table 2). However, in this way, the sulfite ions are not in excess, so when they run out, the sulfur precipitation can permanently reduce the efficiency of the catalyst. Our sacrificial solution contained 35% excess sulfite. This allowed us to monitor the hydrogen evolution until the sulfide ions were completely depleted (Figures S1–S3). In a previous paper, we have already demonstrated that the amount of H_2_ evolved throughout the reaction was equal to the amount of sulfide ions consumed from the solution [48]. It was observed that at the end of the reaction, when the concentration of sulfide ions drops below 0.01 M, the Ni-containing (ZC-xxNi-I) catalysts are always darkened (Figure S2b). A few hours after the illumination is stopped or when the pH of the catalyst suspension is reduced (in the case of aqueous washing), the color of the catalyst reverts to the original yellow color. No similar color change was observed for the Ni-free sample. The color of the catalysts prepared by conventional methods was initially slightly darker, which can be attributed to the precipitation of NiS. At the end of the reaction, a reversible darkening, similar to the above-described case, was also observed for these catalysts, but its intensity was smaller compared to the composite obtained by the similar Ni-impregnation method. In the case of the Ni-free sample, such a darkening was not observed at all. The interpretation of this phenomenon is presented at the end of the characterization (Section 2.2). Notably, the first-time application of the modified (improved) impregnation procedure combined with sulfite excess ensuring the exploitation of the total sulfide amount made us possible to reveal the mechanism of the photocatalytic H_2_ evolution based on the Ni-modified catalyst.

For the most efficient CZS-10Ni-I catalyst, the apparent quantum yield (AQY) was determined by illuminating it with a 415-nm LED. Since the Na_2_S was consumed too quickly when illuminating in the usual way, doubled concentrations of Na_2_S and Na_2_SO_3_ were used for this measurement. The total volume of the illuminated suspension was unchanged at 30 cm^3^ and the amount of catalyst was also the usual 20 mg. The amount of H_2_ evolved was 3.4 mmol/h (170 mmol/h/g) (Figure S4). Considering that the amount of the photons entering the reactor determined by actinometry was 43.0 mmol photons/h, the resulting AQY was 15.8%.

Since the RHP value depends on the light source used, the geometric arrangement and the catalyst concentration, it is not suitable for comparison. It would be much more correct to use AQY as a basis for comparison, but, unfortunately, only the former values are given in many publications. Table 2 summarizes the RHP and AQY values for some Ni-modified CdS-type catalysts. To the best of our knowledge, the RHP value of 170 mmol H_2_/g/h is the highest value published so far for CdS-type catalysts, while the AQY obtained is, if not the highest, certainly good.

### 2.2. Characterization

**Figure 4:**
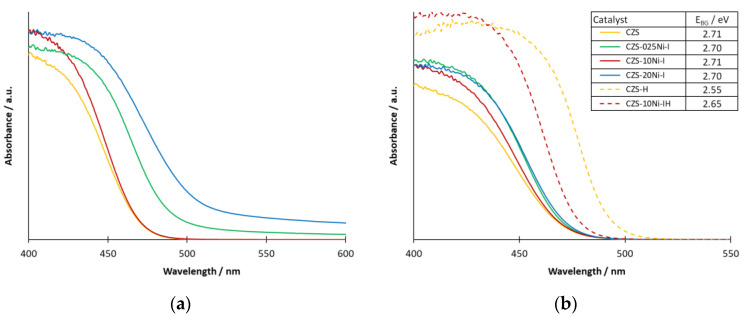
Absorption spectra of catalysts prepared with different methods (**a**) and prepared by impregnation with different amounts of Ni(II) (**b**). The orange curves represent the unmodified CZS catalyst in both (**a**,**b**). The red, blue and green spectra in (**a**) belong to the catalysts CZS-10-I, CZS-10-S, and CZS-10-B, respectively. The green, red, and blue spectra in (**b**) belong to the catalysts CZS-025-I, CZS-10-I, and CZS-20-I, respectively. The dashed curves show the spectra of the corresponding hydrothermally treated catalysts (dashed orange: CZS, dashed red: CZS-10Ni-IH).

The absorption spectra of the CZS-10Ni-x catalysts prepared by the addition of 1% Ni(NO_3_)_2_ in different ways show that the absorption of the impregnated composite is essentially the same as that of the unmodified CZS catalyst (Figure 4a). However, an increase in the spectra of the conventional Ni-modified samples was observed in the whole visible range, which is consistent with the darkening observed during the preparation.

The composites prepared by the impregnation method using various amounts of Ni (CZS-xxNi-I) did not show any significant difference compared to CZS, even at the highest Ni content, while a significant red shift was observed in the absorption edge of the two hydrothermally treated composites (Figure 4b). In the values of BG energies estimated by the Tauc method (SM Figure S5), the hydrothermal treatment caused a decrease of 0.16 eV and 0.06 eV for CZS and CZS-10Ni-I catalysts, respectively. This is consistent with the experience described in [50]. Although a lower BG value may result in more absorbed photons and thus higher catalytic efficiency, as described in our previous paper, a 0.1 eV decrease only results in 5–10% more absorbed photons. Since the change in the efficiency is inconsistent with the change in BG values, we looked for another explanation.

**Figure 5:**
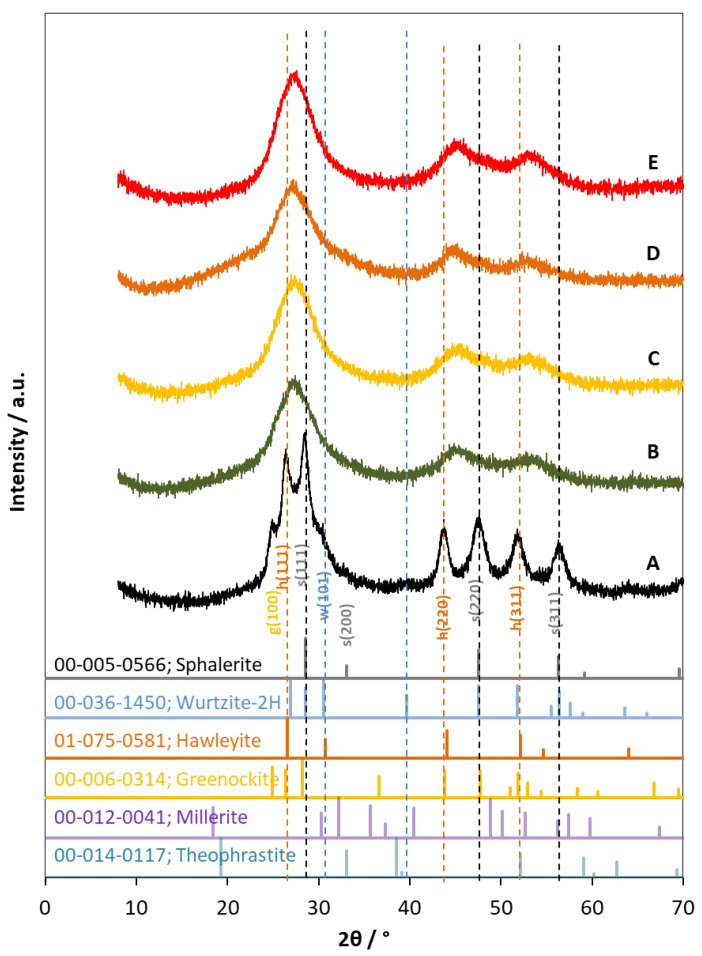
XRD patterns of photocatalysts. (A) reference prepared by a simple mixture of ZnS and CdS. B, C and E are freshly prepared catalysts of CZS (B), CZS-10Ni-I (C), and CZS-10Ni-S (E), and (D) is the pattern of CZS-10Ni-I catalyst after three illumination experiments. The vertical dashed lines designate the characteristic peaks of cubic sphalerite (black), hawleyite (orange), and hexagonal wurtzite (blue).

XRD analyses were taken to determine the phase composition of the catalysts (Figure 5). The pattern of the reference containing a mixture of CdS and ZnS shows well-separated sphalerite and hawleyite peaks. In contrast, for all of the composites examined, only a single peak was detected, with the maximum positions located between the corresponding peaks. The three characteristic peaks at 27.4°, 45.0°, and 53.5° 2θ values can be assigned to the (111), (220), and (311) crystallographic planes, respectively. This suggests that the CdS and ZnS, crystallized in the cubic sphalerite-type lattice, form a solid solution in the prepared products, in which the Zn^2+^ and Cd^2+^ ions substituted each other. The XRD patterns did not change either with Ni-modifications or during illumination.

The investigated composites were not heat-treated, so the crystallite sizes were expected to be small, as can be seen from the TEM images (~5–10 nm). This fact alone confirms the wide bands in the XRD patterns. In addition, both the SEM and TEM elemental maps show a slight inhomogeneity of the composites, suggesting that the detected peaks are the result of the overlapping of several peaks close to each other. This also leads to a widening of the peaks and means that the Scherrer equation is not applicable for the determination of the real crystallite size in this case. Although the presence of an amorphous phase cannot be excluded, the patterns are characteristic of a typical nanocrystalline structure.

**Figure 6:**
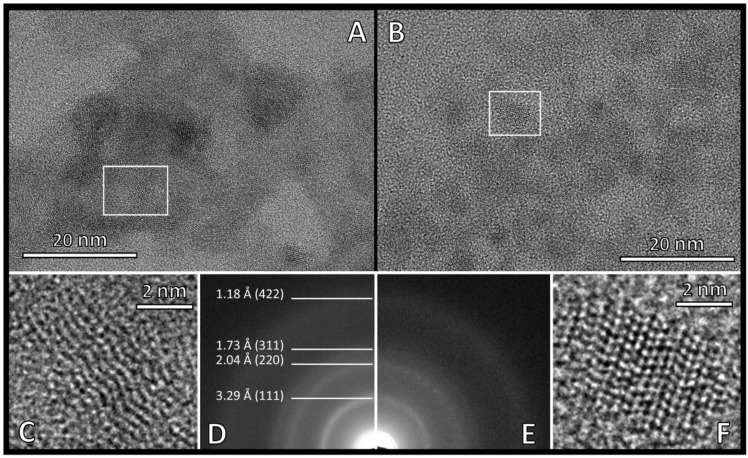
HRTEM images (**A**,**B**), its magnified details illustrating the twin boundaries (**C**,**F**) and selected area diffraction ring patterns (SAED) (**D**,**E**) of CZS (left: **A**,**C**,**D**) and CZS-10Ni-I (right: **B**,**E**,**F**) catalysts.

It is clearly seen from the HRTEM images of CZS and CZS-10Ni-I composites (Figure 6A,B) that Ni-modification does not noticeably change the structure of the composites. In both cases, aggregates of ~5–10 nm of individual uniform crystallites can be observed. The composites dominantly crystallize in a sphalerite lattice, as confirmed by the d-values determined from SAED rings (3.29 Å (111), 2.04 Å (220), 1.73 Å (311), and 1.18 Å (422)) (Figure 6D,E). After Ni-modification, the sphalerite structure of the original CZS catalyst is not changed, and the crystal parameters determined from the SAED rings are not altered either, besides, the ring broadening is similar. These results are consistent with the conclusions drawn from the XRD images and also confirm that the crystallite size does not change. For both composites, it is noted that sphalerite-wurtzite twin boundaries are present (Figure 6C,F), but they are not common. The low catalytic activity of the CZS catalyst and the low number of twin boundaries in its structure are in agreement with previous findings [24,27,28] that the high efficiency of CdS-ZnS catalysts is due to the high number of twin boundaries. At the same time, it also implies that the high efficiency of Ni-modified catalyst is a result of other factors.

**Figure 7:**
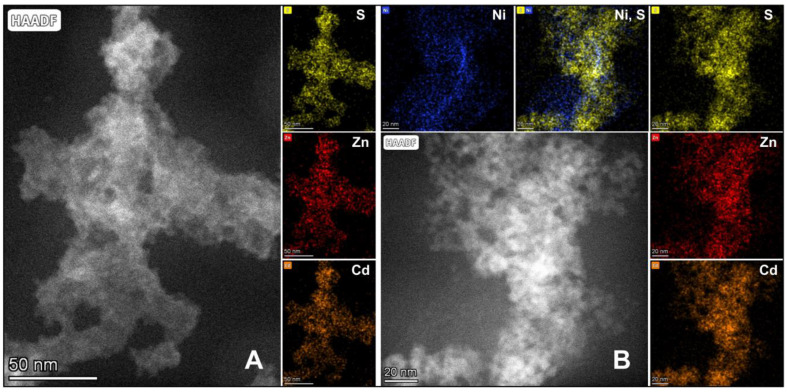
STEM composite images of catalysts CZS (**A**) and CZS-10Ni-I (**B**) created from the HAADF signal and the elemental maps.

The STEM elemental maps of the CZS and CZS-10Ni-I catalysts (Figure 7A,B) show that the distribution of Cd, Zn, and S is nearly homogeneous. Although a slight Cd-Zn inhomogeneity is observed, neither pure CdS- nor pure ZnS-containing areas were found. In the most cadmium-rich or zinc-rich areas, the ratio was not more extreme than 30–70%. This suggests that CdS and ZnS form a solid solution. Ni is difficult to find in the composite due to its low concentration in the CZS-10Ni-I catalyst, so it can be studied mainly in those parts where it is slightly enriched. In these cases, Ni was observed on the surface of CZS or eventually in small areas enclosed by CZS grains. No Ni was found in isolated islands, which also suggests that the size of Ni-containing particles is very small, even forming clusters. In Ni-rich areas, sulphur can only be found close to the limit of detection, suggesting that Ni(II) is not present as NiS. In contrast, the surface-modified product (CZS-10Ni-S, SM Figure S6) contained a significant number of NiS-containing grains.

In order to determine the more accurate composition, SEM elemental maps of the CZS and CZS-10Ni-I catalysts were also taken (SM Figure S7), where the elemental composition was obtained from the average of an area of 400 × 500 μm^2^. The results are summarized in Table 3. For both catalysts investigated, both the Zn/Cd ratio and the ratio of sulfur to total metal content are in agreement with the theoretical ratio within error limits. In the CZS-10Ni-I catalyst, the ratio of Ni to total Zn and Cd is also in agreement with the expected value, but due to its large error, this was checked by ICP-OES measurement. According to this, 45% of the amount of the Ni(II) added to the catalyst was bound, so the calculated Ni content relative to the total amount of Zn and Cd is (n_Ni_/(n_Cd_ + n_Zn_)) 0.45% (i.e., taking the sulphur content into account) the Ni atomic% is 0.225%.

Surface composition was examined by XPS, before and after irradiation of a CZS-10Ni-I sample. Since the catalyst, which was dark after irradiation, turned back to yellow during washing, the final phase of irradiation was simulated immediately before the XPS measurement. For this purpose, a drop-casted suspension of CZS-10Ni-I, in 100 μL aqueous Na_2_SO_3_ (2.5 mg) was irradiated for 30 min on a transparent indium tin oxide (ITO) glass slide using a 300-W Xe lamp in the wavelength range 360–600 nm at 100 mW/cm^2^ light intensity (MAX-303 fiber optic lamp, produced by the ASAHI Co., Japan). Another sample was left as-prepared, without irradiation, and both suspensions were dried under air at 80 °C for 20 min. The irradiated sample visibly changed its color to grey-black (note that after several hours the original yellow color returned upon standing).

The Cd, Zn, S, and Ni surface atomic concentrations (%) obtained by XPS of both the as-prepared and the irradiated samples corroborated with the elemental composition calculated from EDS (Table 3). In addition, the chemical environment of the elements was scrutinized to reveal if irradiation caused any changes in the (catalytically most relevant) surface layer. However, XPS confirmed that both Zn and Cd are present as sulfides before and after irradiation, based on the unchanged Zn 2p and Cd 3d doublets and their typical binding energies (in Figures S8 and S9 we show representative spectra) [51,52]. CdS could be also identified from the Auger parameter (AP-3d^5/2^, M5N45N45) of 779.86 eV (the literature value for CdS is 779.8 eV [53]).

The lower sulphur-metal ratio obtained from XPS analysis is consistent with the finding of Li et al. that sulphur species on the surface of the CdS-ZnS composite are substituted by oxygen species [54]. The higher Zn:Cd ratio compared to the bulk suggests that the ZnS is deposited more slowly during the CdS-ZnS co-precipitation, resulting in a slight enrichment at the surface.

**Figure 8:**
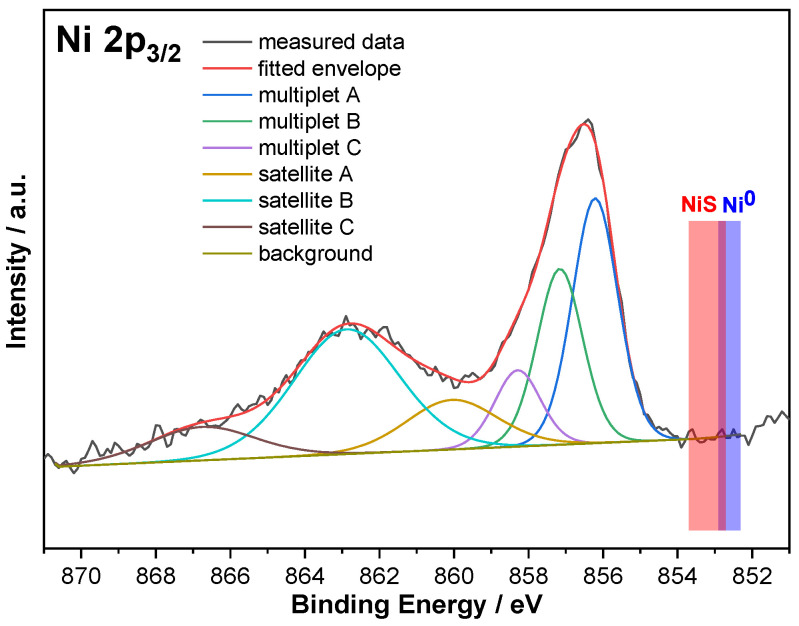
High-resolution Ni 2p_3/2_ XP spectrum of CZS-10Ni-I catalyst after its irradiation. The regions highlighted in red and blue denote the typical binding energies for NiS and Ni^0^ phases, respectively, that are absent from our samples.

In our attempt to interpret the role of Ni in photocatalysis, and in conjunction, the reason for the reversible darkening—we suspected to originate from the occurrence of Ni(III)—we investigated in detail the Ni 2p_3/2_ binding energy region after irradiation (Figure 8). Based on the Ni 2p binding energy, Ni is predominantly in the +2 oxidation state and the presence of metallic Ni and a NiS phase could be unequivocally excluded (Figure 8, the expected binding energy regions are highlighted in red and blue for Ni^0^ and NiS, respectively) [55]. The presence of NiO is also unlikely since its multiplet signal at lower binding energies relative to those measured is missing [55]. The Ni 2p_3/2_ signal and its satellite could be best fitted with multiplets matching Ni(OH)_2_ [55] (Figure 8). Last, in the irradiated sample the presence of a low amount of Ni(III)—having similar binding energies to Ni(II)—cannot be excluded (nor confirmed), but due to the a priori low Ni content of the catalyst (<1%) a conclusive statement cannot be made.

**Figure 9:**
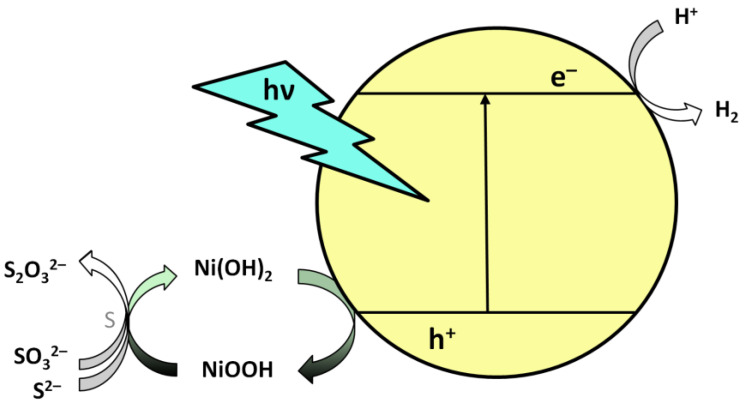
Schematic illustration of the proposed mechanism of photocatalytic hydrogen production by Ni(OH)_2_ modified CZS catalysts.

HR-TEM and XPS measurements confirmed that Ni is mainly present in the Ni(OH)_2_ form on the catalyst. This information, combined with the darkening observed in Ni-containing catalysts, helps to propose a new mechanism that is different from the usual one. Since the Ni-free sample was not darkened, the blackening may be due to a Ni compound. The black compounds in this system may be metal Ni, NiS, and NiOOH. The formation of metal Ni would be expected to be irreversible and it would not be reasonable to expect the color change to occur only at the end of the reaction. If NiS were the cause of the change, it would be expected to occur at the beginning of the reaction, at the highest sulfide concentration, rather than several hours later when the sulfide concentration drops significantly. The presence of NiOOH as an intermediate is reasonable since Ni(OH)_2_ bound on the catalyst surface can be oxidized by the photogenerated holes. Due to its strong oxidizing character, NiOOH reacts with sulfide ions in solution, thus reverting back to pale green Ni(OH)_2_. This may cause NiOOH to be unable to accumulate as long as the sulfide concentration is high, but it is allowed to accumulate as it is depleted. It is also well-known that NiOOH is not stable in aqueous media, especially if the solution is not strongly basic, due to its strong oxidizing character. According to this hypothesis, we assume the mechanism shown in Figure 9, in which processes (1–5) may take place during illumination.

(1) $$CZS+h\nu \to e^{-}+h^{+}$$

(2) $$2H_{2}O+{2e}^{-}\to H_{2}+2OH^{-}$$

(3) $$Ni{\left(OH\right)}_{2}+OH^{-}+h^{+}\to NiOOH+H_{2}O$$

(4) $$2NiOOH+S^{2-}+2H_{2}O\to 2Ni{\left(OH\right)}_{2}+S+2OH^{-}$$

(5) $$SO_{3}^{2-}+S\to S_{2}O_{3}^{2-}$$

The role of Ni(II) co-catalysts is usually attributed to the trapping of CB electrons, whereas in our suggestion Ni(OH)_2_ assists in the catalytic reaction not on the reduction but on the oxidation side by catalyzing the oxidation of the sulfide sacrificial agent. This mechanism proposes that Ni(OH)_2_ acts as a hole trap on the surface of the CdS-ZnS catalyst, enhancing charge separation and thus reducing the probability of electron-hole recombination. In the case of catalysts used for photocatalytic H_2_ evolution, no reference was found to either the appearance of NiOOH species or the possible hole-trapping role of Ni(OH)_2_. However, the formation of NiOOH has been pointed out by several authors when using Ni(OH)_2_ catalyst or co-catalyst for oxidation processes [56,57,58]. To complete the catalytic cycle, the thiosulfate needs to be converted back to elemental sulphur and sulfite, the conditions of which have been studied in detail by Linkous et al. [59].

## 3. Materials and Methods

### 3.1. Materials

Zinc acetate dihydrate (Zn(CH_3_COO)_2_⋅2H_2_O), cadmium acetate dihydrate (Cd(CH_3_COO)_2_⋅2H_2_O), nickel(II) nitrate hexahydrate (Ni(NO_3_)_2_⋅6H_2_O) and sodium sulfite (Na_2_SO_3_) were purchased from Reanal (Budapest, Hungary), and sodium sulfide nonahydrate >98% (Na_2_S⋅9H_2_O) from Merck (Darmstadt, Germany). The water applied was cleaned by a Millipure Elix equipment completed with a Milli-Q 50 purification system (Millipore S.A.S., Molsheim, France). The solutions containing sulfide/sulfite were prepared in advance by using argon-bubbled Milli-Q water and kept in the freezer in pre-measured portions for further experiments.

### 3.2. Preparation of Photocatalysts

#### 3.2.1. Unmodified Cd_0.5_Zn_0.5_S Catalyst (CZS and CZS-H)

3 mmol cadmium acetate dihydrate and 3 mmol zinc acetate dihydrate were dissolved in 25 mL Milli-Q water, 4.5 mL of 25% ammonia solution was added, and the mixture was stirred for 10 min while Ar gas was bubbled through it. 6.6 mmol (10% excess) of Na_2_S⋅9H_2_O was dissolved in 10 mL Milli-Q water which was deaerated by bubbling of argon gas for 20 min. This Na_2_S solution was added to the solution containing the metal ions under vigorous stirring. The light yellow precipitate formed immediately. This suspension was stirred for another 30 min with further Ar bubbling. The precipitate was washed 3 times with Milli-Q water and centrifuged. The catalysts were then stored in aqueous suspensions. The stability of the catalyst during this type of storage was described in our previous article [50]. It was found that the activity of the catalyst is almost unchanged even after 1 year of storage. This catalyst is abbreviated by “CZS”.

The CZS-H catalyst was prepared in a similar way, but before the washing procedure the suspension was poured into a 50-mL Teflon-lined autoclave and hydrothermally treated at 170 °C for 3 h.

The elemental composition of both catalysts was tested by SEM-EDS elemental analysis, and it was found that the measured Zn:Cd ratio proved to be 1.0 ± 0.05. The supernatant after the first and second washing cycles was also checked by ICP, and neither Zn nor Cd was detected in it. After the third centrifugation the sulfide content of the supernatant was always tested by 0.1 M Pb(NO_3_)_2_ solution. For the Ni modification by impregnation method, it is very important that the catalyst to be modified should be sulfide-free.

#### 3.2.2. Ni-Modified Cd_0.5_Zn_0.5_S Catalysts (CZS-xxNi-y and CZS-10Ni-IH)

Three different methods were used to prepare Ni-modified catalysts. In the first case, we followed the procedure described for the preparation of the CZS catalyst, except that 0.06 mmol Ni(NO_3_)_2_⋅6H_2_O (1.0% of the sum of Zn and Cd content) was added to the solution of metal acetates and the precipitation was performed with 6.67 mmol (10% excess) of Na_2_S⋅9H_2_O. The catalyst was further processed, purified, and stored as described above. The resulting bulk-modified catalyst was designated as “CZS-10Ni-B”.

The second method was the best-known and most commonly used in the literature. A CZS catalyst suspension containing 0.5 mmol (60 mg) of Cd_0.5_Zn_0.5_S was diluted to 10 mL with Milli-Q water, to which 0.5 mL of 25% NH_4_OH solution and 0.05 mL of 0.1 M Ni(NO_3_)_2_ solution (0.005 mmol) were added (1.0% of the sum of Zn and Cd content). After 10 min stirring, 0.0055 mmol Na_2_S dissolved in 1 mL of water was added (10% excess). The precipitate immediately turned dark yellow. After a further 30 min stirring, the further purification procedures were the same as described for the CZS catalyst. The resulting surface-modified catalyst was designated as “CZS-10Ni-S”.

For the impregnation procedure, 0.5 mmol of CZS catalyst was washed until the supernatant was completely sulfide-free. The thoroughly washed CZS catalyst was then suspended in 10 mL of water, to which 0.5 mL of 25% NH_4_OH solution and then the appropriate amount of Ni(NO_3_)_2_ solution (0.125%, 0.25%, 0.5%, 1.0% or 2.0% relative to the total amount of Zn and Cd) was added. In contrast to the conventional surface modification procedure, this suspension was left to stand overnight after 30 min of mixing, centrifuged twice, washed with Milli-Q water, and only afterward Na_2_S was added in 10% excess relative to the original Ni content during vigorous mixing. It was stirred for a further 30 min while Ar gas was bubbled through it. Subsequent processing was as described for the CZS catalyst. The resulting catalysts were designated as CZS-xxNi-I, where xx = 0125, 025, 05, 10, or 20, depending on the amount of Ni used (i.e., 0.125%, 0.25%, 0.5%, 1.0%, or 2.0%), respectively, relative to the total amount of Zn and Cd. The color of the modified catalyst remained yellow, as the original CZS. It did not darken even at 2% Ni content.

The CZS-10Ni-IH catalyst was prepared similarly to CZS-10Ni-I, except that instead of leaving the Ni(II) containing suspension to stand overnight, it was hydrothermally treated at 170 °C for 3 h.

### 3.3. Characterization of Photocatalysts

The phase composition was determined by X-ray diffraction measurement (Philips PW3710, Cu Kα radiation, 50 kV and 40 mA) (Philips Analytical B.V., Almelo, The Netherlands). Data collections were carried out with an X’Pert Data Collector software (2.0e, PANalytical B.V., Almelo, the Netherlands, 2010). HighScore Plus software (5.0, Malvern Panalytical B.V., Almelo, the Netherlands, 2021) was used for phase identification. The 01–075-0581, 00-005-0566, 00-006-0314, and 00-036-1450 Powder Diffraction File (PDF, PDF-2 2021) of ICDD (International Centre for Diffraction Data) of hawleyite, sphalerite, greenockite, and wurtzite, respectively, was used to identify phases. The samples were prepared by drying the catalyst suspension on a glass plate.

A Specord S600 spectrofluorometer with an integrating sphere (Analytik Jena GmbH + Co., Jena, Germany) was used to measure the diffuse reflectance spectra, from which the absorption spectra of the catalysts were calculated using the Kubelka-Munk function [60] and the band gap values were determined using the Tauc method [61].

Samples for transmission electron microscopy (TEM) were prepared by depositing a drop of a diluted aqueous suspension of the original samples on copper TEM grids covered by continuous carbon amorphous support film. TEM analyses were performed using a Talos F200X G2 instrument (Thermo Fisher Scientific, Walthan, MA, USA), operated at 200 kV accelerating voltage, equipped with a field-emission gun and a four-detector Super-X energy-dispersive X-ray spectrometer, and capable of working in both conventional TEM and scanning transmission (STEM) modes. In our study, TEM bright-field images, HRTEM images, and STEM high-angle annular dark-field (HAADF) images were collected to visualize the crystal size and the morphology of the particles, HRTEM images as well as electron diffraction patterns were used to study the structural properties, and STEM-EDS elemental maps were collected to measure and visualize the chemical compositions. The morphologies of the catalysts were also studied by Schottky-field emission scanning electron microscope (SEM) (FEI/ThermoFisher, Waltham, MA, USA, Apreo S). The energy-dispersive-spectroscopy (EDS) studies were performed by EDAX Ametek equipped with Octane Elect Plus detector.

Ni-, Zn-, and Cd-content were determined by means of the ICP-OES technique. Measurements were carried out in a Spectroflame Modula E type (Spectro Analytical Instruments GmbH, Kleve, Germany) spectrometer. The spectrometer is equipped with a horizontal torch and axial plasma viewing. The calibration curves were prepared using a three-point standard solution series: in 0, 2, and 8 mg/L concentration for Ni, and a 4-point standard solution series: in 0, 1, 2, and 4 mg/L concentration for Zn and Cd. Ni, Zn, and Cd were measured at 231.604 nm, 213.856 nm, and at 228.802 nm emission lines, respectively. Detection limits were 0.007 mg/L for Ni, 0.008 for Zn, and 0.003 mg/L for Cd.

To determine the composition and chemical state of the sample surfaces, XPS measurements were performed in a Thermo Scientific ESCALAB Xi+ instrument (Assembled in Czech Republic, Brno). Monochromatized Al K-alpha source (1486.6 eV), with a 200 µm spot size was used. On each sample, wide-range spectra were collected at an analyzer pass energy of 80 eV, for surveying the elemental composition. For quantitative and chemical state analysis, high-resolution spectra were recorded at 20 eV pass energy for the following photoelectron lines: C 1s, Cd 3d, Zn 2p, O 1s, Ni 2p, and S 2p regions. The charging of the sample surface was compensated by using the automatic built-in charge compensation system. The energy scale was adjusted to obtain the adventitious C 1s spectral component binding energy of 284.8 eV.

### 3.4. Photochemical Experiments

The photochemical experiments were performed under the conditions described by Fodor et al. [48], in a double-necked reactor of 40 cm^3^ volume (14 mm height, 60 mm diameter). The total volume of all sacrificial solutions was 30 cm^3^ and they contained 0.145 M Na_2_S, 0.19 M Na_2_SO_3_, and 20 mg of catalyst (0.67 g/dm^3^). Before illumination all samples were deaerated by argon bubbling for 10 min. Two 7 W 6000 K Optonica visible LEDs (Sofia, Bulgaria) were used as light source. For the determination of the apparent quantum yield (6), a 415-nm LED with 13 nm full width at half maximum (fwmh) was used. Its intensity was determined by trioxalato-ferrate(III) actinometer. The amount of photons incident on the reactor was found to be 43.0 mmol/h (light intensity: 125 mW/cm^2^). The emission spectra of these light sources are depicted in the SM (Figure S10).

(6) $$AQY=\frac{The\;number\;of\;reacted\;electrons}{The\;number\;of\;incident\;photons}=\;\frac{2\cdot the\;number\;of\;evolved\;H_{2}\;molecules}{The\;number\;of\;incident\;photons}$$

The evolved hydrogen was bubbled into a vessel filled with 1 mM NaOH solution and its volume was calculated from the mass of the NaOH solution displaced. Mass data were collected every minute by using a Kern PCB balance (D-72336 Balingen, Germany) connected to a PC. The illuminations were always performed until the end of the reactions. The system was always checked for gas tightness before the irradiation started. The experiments were started at room temperature, but the photoreactor was not thermostated. The temperature changes in the reactor have been reported in our previous article [50]. In all cases, the temperature stabilized at 42–45 °C within 45–60 min. This, of course, affects the rate of H_2_ evolution, so when calculating its average, the first 1 h was ignored. In all cases, the amount of H_2_ evolved by the end of the reaction was equal to the amount of Na_2_S measured (4.4 mmol, ~105 mL). The average RHP value was calculated consistently from the times required for the production of 20 and 50 mL H_2_ since H_2_ evolution showed a steady rate for all our systems in this range. Time data required to calculate RHP values are given in Table S1 in the SM. The photochemical reaction in sacrificial solutions containing either no catalyst or pure ZnS instead of our catalyst was also tested. After reaching thermal equilibrium, no displaced solution was detected (i.e., no hydrogen evolution was observed (see Figure S11 and the related text in the SM)). Based on performing at least three parallel illuminations, the error of the calculated average RHP values was evaluated to be 25 μmol/h. It is important to note that the reaction mixture was not stirred during irradiation, as we wanted to approach the conditions for possible industrial application.

## 4. Conclusions

Ni(II)-modification of Cd_0.5_Zn_0.5_S composites by impregnation, which is a simple but unconventional method for this type of catalyst, proved to be appreciably efficient for visible-light-driven photocatalytic hydrogen generation from sulfide-containing aqueous system. Modification with 1% Ni(II) led to the highest quantum yield of H_2_ evolution (15.8% at 415-nm irradiation). The impregnated catalyst, in addition to the enhanced efficiency, also showed a high photostability, with no decrease in the activity below the initial value after three consecutive illuminations. On the basis of the results of XPS analysis, Ni(II) was found in the catalyst as Ni(OH)_2_, which is, according to the observations during the illuminations, oxidized to NiOOH, which is a transitional form of nickel in this system. These observations were made possible by the first-time combination of the improved impregnation (as an unconventional method for Ni-modification) and the sulfite excess in the sacrificial electron donor solution (for H_2_ evolution). Based on these, a mechanism was proposed in which Ni(OH)_2_ plays the role of a hole trap and also a co-catalyst in the oxidation of sulfide ions. This role, through the formation of NiOOH as an intermediate, was suggested for the first time in photocatalytic hydrogen-producing systems based on Ni-modified CdS-ZnS catalysts. These results may contribute to the further development of such systems toward practical application for solar energy conversion and storage in hydrogen as an environmentally friendly fuel.

## Figures and Tables

**Table 1:** Labels and key conditions for the applied catalysts.

Label	Ni% ^1^	Method	Hydrothermal Treatment
CZS	0	–	–
CZS-H	0	–	170 °C, 3 h
CZS-10Ni-S	1.0%	surface modification	–
CZS-10Ni-B	1.0%	bulk modification	–
CZS-0125Ni-I	0.125%	impregnation	–
CZS-025Ni-I	0.25%	impregnation	–
CZS-05Ni-I	0.5%	impregnation	–
CZS-10Ni-I	1.0%	impregnation	–
CZS-10Ni-IH	1.0%	impregnation	170 °C, 3 h
CZS-20Ni-I	2.0%	impregnation	–

^1^ Amount of added Ni(II) relative to the total amount of Cd(II) and Zn(II) (Ni% = n_Ni_/(n_Cd_ + n_Zn_)).

**Table 2:** Summary of the efficiency of some Ni-modified Cd_x_Zn_1–x_S catalysts and the content of Na_2_S and Na_2_SO_3_ in the sacrificial solution.

x/NiX% ^1^	Preparation Method ^2^	RHP (mmol H_2_/g/h)	AQY%	c_Na2S_, c_Na2SO3_ (mol/dm^3^)	Reference
0.5/0.025% NiS	S + Ph	1.4 ^3^	33.9%	0.35, 0.25	[25]
0.5/0.25% NiS	S + Ph	38.2	not given	0.35, 0.25	[27]
0.4/0.1% NiS	S + Ph	1.2	not given	0.1, 0.1	[34]
1/5% NiS	S	1.13	6.1%	0.35, 0.25	[45]
1/5% NiS	B	49.2	74.6	50% lactic acid	[23]
1/5% NiS	B	37.2	not given	0.35, 0.25	[23]
1/5% NiS	S	24	not given	0.35, 0.25	[23]
1/2.7% Ni_x_S_y_	enzymatic	10.5	not given	0.1, 0.1	[49]
0.25/0.1% NiS	S	110	14.9	0.117, 0.16	[50]
0.5/1% Ni(OH)_2_	I	170	15.8	0.29, 0.38	this work

^1^ x is the Cd ratio in Cd_x_Zn_1–x_S, the Ni% are given as Ni% = n_Ni_/(n_Cd_ + n_Zn_); ^2^ B: “bulk” modification (co-precipitation), S: surface modification, Ph: photocatalytic deposition, I: impregnation; ^3^ The RHP was given only in mmol H_2_/h unit.

**Table 3:** Elemental composition of CZS and CZS-10Ni-I catalysts calculated from SEM-EDS and XPS analysis. Elemental contents are given in atomic%.

	CZS	CZS-10Ni-I	CZS	CZS-10Ni-I	CZS-10Ni-I after Illumination
Element	from SEM/EDS			from XPS	
Zn	22.8 ± 0.8%	25.4 ± 0.7%	33.7 ± 1.9%	33.1 ± 0.6%	31.2 ± 2.0%
Cd	23.0 ± 0.6%	23.8 ± 0.5%	19.9 ± 0.8%	20.6 ± 0.4%	22.4 ± 0.4%
S	54.2 ± 2.3%	50.6 ± 2.1%	46.4 ± 1.5%	46.0 ± 0.9%	46.1 ± 0.5%
Ni	–	0.22 ± 0.09%	–	0.32 ± 0.04%	0.32 ± 0.04
Zn/Cd ^1^	0.99 ± 0.05	1.07 ± 0.04	1.69 ± 0.10	1.60 ± 0.06	1.40 ± 0.12
1000 × Ni/(Zn + Cd) ^1^	–	4.5 ± 1.8	–	6.0 ± 0.8	5.9 ± 0.8
S/(Zn + Cd) ^1^	1.19 ± 0.05	1.03 ± 0.04	0.864 ± 0.030	0.856 ± 0.017	0.860 ± 0.10

^1^ Elemental ratio.

## Data Availability

The data presented in this study are available on request from the corresponding author. The data are not publicly available due to privacy.

## Supplementary Materials

The supporting information can be downloaded at: https://www.mdpi.com/article/10.3390/ijms24129802/s1.

## References

[1] Matsuoka M., Kitano M., Takeuchi M., Tsujimaru K., Anpo M., Thomas J.M. (2007). Photocatalysis for new energy production. Catal. Today.

[2] Zhang Y., Heo Y.-J., Lee J.-W., Lee J.-H., Bajgai J., Lee K.-J., Park S.-J. (2018). Photocatalytic Hydrogen Evolution via Water Splitting: A Short Review. Catalysts.

[3] Acar C., Dincer I., Zamfirescu C. (2014). A review on selected heterogeneous photocatalysts for hydrogen production. Int. J. Energy Res..

[4] Kudo A. (2006). Development of photocatalyst materials for water splitting. Int. J. Hydrogen Energy.

[5] Kudo A., Miseki Y. (2009). Heterogeneous photocatalyst materials for water splitting. Chem. Soc. Rev..

[6] Dan M., Yu S., Li Y., Wei S., Xiang J., Zhou Y. (2020). Hydrogen sulfide conversion: How to capture hydrogen and sulfur by photocatalysis. J. Photochem. Photobiol. C Photochem. Rev..

[7] Zhang K., Guo L. (2013). Metal sulphide semiconductors for photocatalytic hydrogen production. Catal. Sci. Technol..

[8] Zhu J., Zäch M. (2009). Nanostructured materials for photocatalytic hydrogen production. Curr. Opin. Colloid Interface Sci..

[9] Lu Q., Yu Y., Ma Q., Chen B., Zhang H. (2016). 2D Transition-Metal-Dichalcogenide-Nanosheet-Based Composites for Photocatalytic and Electrocatalytic Hydrogen Evolution Reactions. Adv. Mater..

[10] Guo L., Jing D., Liu M., Chen Y., Shen S., Shi J., Zhang K. (2014). Functionalized nanostructures for enhanced photocatalytic performance under solar light. Beilstein J. Nanotechnol..

[11] Joe J., Yang H., Bae C., Shin H. (2019). Metal chalcogenides on silicon photocathodes for efficient water splitting: A mini overview. Catalysts.

[12] Lyubina T.P., Kozlova E.A. (2012). New photocatalysts based on cadmium and zinc sulfides for hydrogen evolution from aqueous Na_2_S-Na_2_SO_3_ solutions under irradiation with visible light. Kinet. Catal..

[13] Xing C., Zhang Y., Yan W., Guo L. (2006). Band structure-controlled solid solution of Cd_1–x_Zn_x_S photocatalyst for hydrogen production by water splitting. Int. J. Hydrogen Energy.

[14] Koca A. (2002). Photocatalytic hydrogen production by direct sun light from sulfide/sulfite solution. Int. J. Hydrogen Energy.

[15] Chan C.-C., Chang C.-C., Hsu C.-H., Weng Y.-C., Chen K.-Y., Lin H.-H., Huang W.-C., Cheng S.-F. (2014). Efficient and stable photocatalytic hydrogen production from water splitting over Zn_x_Cd_1–x_S solid solutions under visible light irradiation. Int. J. Hydrog. Energy.

[16] Li X., Xue F., Li N., Wei X., Liu H., Zhou J., Lyu B., Liu M. (2020). One-Pot Hydrothermal Synthesis of MoS_2_/Zn_0.5_Cd_0.5_S Heterojunction for Enhanced Photocatalytic H_2_ Production. Front. Chem..

[17] De G.C., Roy A.M., Bhattacharya S.S. (1996). Effect of n-Si on the photocatalytic production of hydrogen by Pt-loaded CdS and CdS/ZnS catalyst. Int. J. Hydrog. Energy.

[18] Stroyuk A.L., Raevskaya A.E., Korzhak A.V., Kotenko I.E., Glebov E.M., Plyusnin V.F., Kuchmii S.Y. (2009). Photocatalytic production of hydrogen in systems based on Cd_x_Zn_1–x_S/Ni^0^ nanostructures. Theor. Exp. Chem..

[19] Liu X., Liang X., Wang P., Huang B., Qin X., Zhang X., Dai Y. (2017). Highly efficient and noble metal-free NiS modified Mn_x_Cd_1–x_S solid solutions with enhanced photocatalytic activity for hydrogen evolution under visible light irradiation. Appl. Catal. B Environ..

[20] Zhai H., Liu X., Wang P., Huang B., Zhang Q. (2018). Enhanced photocatalytic H_2_ production of Mn_0.5_Cd_0.5_S solid solution through loading transition metal sulfides XS (X = Mo, Cu, Pd) cocatalysts. Appl. Surf. Sci..

[21] Saraswathi Amma B., Manzoor K., Ramakrishna K., Pattabi M. (2008). Synthesis and optical properties of CdS/ZnS coreshell nanoparticles. Mater. Chem. Phys..

[22] Wang Z., Li M., Li J., Ma Y., Fan J., Liu E. (2022). NiS_x_ modified Mn_0.5_Cd_0.5_S twinned homojunctions for efficient photocatalytic hydrogen evolution. J. Environ. Chem. Eng..

[23] He K., Guo L. (2017). NiS modified CdS pyramids with stacking fault structures: Highly efficient and stable photocatalysts for hydrogen production from water. Int. J. Hydrogen Energy.

[24] Mersel M.A., Fodor L., Pekker P., Jakab M., Makó É., Horváth O. (2021). Effects of preparation conditions on the efficiency of visible-light-driven hydrogen generation based on Cd_0.25_ Zn_0.75_S photocatalysts. Catalysts.

[25] Li N., Zhou B., Guo P., Zhou J., Jing D. (2013). Fabrication of noble-metal-free Cd0.5Zn0.5S/NiS hybrid photocatalyst for efficient solar hydrogen evolution. Int. J. Hydrog. Energy.

[26] Qin Z., Xue F., Chen Y., Shen S., Guo L. (2017). Spatial charge separation of one-dimensional Ni_2_P-Cd_0.9_Zn_0.1_S/g-C_3_N_4_ heterostructure for high-quantum-yield photocatalytic hydrogen production. Appl. Catal. B Environ..

[27] Chen M., Wu P., Zhu Y., Yang S., Lu Y., Lin Z. (2018). Enhanced photocatalytic H_2_ production activity of CdZnS with stacking faults structure assisted by ethylenediamine and NiS. Int. J. Hydrog. Energy.

[28] Liu M., Jing D., Zhou Z., Guo L. (2013). Twin-induced one-dimensional homojunctions yield high quantum efficiency for solar hydrogen generation. Nat. Commun..

[29] Huang H.-B., Fang Z.-B., Yu K., Lü J., Cao R. (2020). Visible-light-driven photocatalytic H_2_ evolution over CdZnS nanocrystal solid solutions: Interplay of twin structures, sulfur vacancies and sacrificial agents. J. Mater. Chem. A.

[30] Dong W., Liu Y., Zeng G., Zhang S., Cai T., Yuan J., Chen H., Gao J., Liu C. (2018). Regionalized and vectorial charges transferring of Cd_1–x_Zn_x_S twin nanocrystal homojunctions for visible-light driven photocatalytic applications. J. Colloid Interface Sci..

[31] Zhao X., Luo Z., Hei T., Jiang Y. (2019). One-pot synthesis of Zn_x_Cd_1–x_S nanoparticles with nano-twin structure. J. Photochem. Photobiol. A Chem..

[32] Li Y., Sun B., Lin H., Ruan Q., Geng Y., Liu J., Wang H., Yang Y., Wang L., Chiu Tam K. (2020). Efficient visible-light induced H_2_ evolution from T-Cd_x_Zn_1–x_S/defective MoS_2_ nano-hybrid with both bulk twinning homojunctions and interfacial heterostructures. Appl. Catal. B Environ..

[33] Sun Y., Xu C., Ma H., Li G., Chen L., Sun Y., Chen Z., Fang P., Fu Q., Pan C. (2021). Synthesis of flower-liked twin crystal ternary Ni/NiS/Zn_0.2_Cd_0.8_S catalyst for highly efficient hydrogen production. Chem. Eng. J..

[34] Wang J., Li B., Chen J., Li N., Zheng J., Zhao J., Zhu Z. (2012). Enhanced photocatalytic H_2_-production activity of Cd_x_Zn_1–x_S nanocrystals by surface loading MS (M = Ni, Co, Cu) species. Appl. Surf. Sci..

[35] Gan S., Deng M., Hou D., Huang L., Qiao X., Li D. (2021). An amorphous NiS x film as a robust cocatalyst for boosting photocatalytic hydrogen generation over ultrafine ZnCdS nanoparticles. Mater. Adv..

[36] Guan S., Fu X., Zhang Y., Peng Z. (2018). β-NiS modified CdS nanowires for photocatalytic H_2_ evolution with exceptionally high efficiency. Chem. Sci..

[37] Zhou X., Sun H., Zhang H., Tu W. (2017). One-pot hydrothermal synthesis of CdS/NiS photocatalysts for high H_2_ evolution from water under visible light. Int. J. Hydrog. Energy.

[38] Lingampalli S.R., Roy A., Ikram M., Rao C.N.R. (2014). Visible-light induced hydrogen generation with ZnO/NiO/Cd_1−x_Zn_x_S (x = 0.0, 0.2) heterostructures. Chem. Phys. Lett..

[39] Xu Y., Gong Y., Ren H., Liu W., Li C., Liu X., Niu L. (2018). Insight into enhanced photocatalytic H_2_ production by Ni(OH)_2_-decorated Zn_x_Cd_1–x_S nanocomposite photocatalysts. J. Alloys Compd..

[40] Zhang L., Wang G., Jin Z. (2019). Growth of Zn_0.5_Cd_0.5_S/α-Ni(OH)_2_ heterojunction by a facile hydrothermal transformation efficiently boosting photocatalytic hydrogen production. New J. Chem..

[41] Markovskaya D.V., Kozlova E.A., Gerasimov E.Y., Bukhtiyarov A.V., Kozlov D.V. (2018). New photocatalysts based on Cd_0.3_Zn_0.7_S and Ni(OH)_2_ for hydrogen production from ethanol aqueous solutions under visible light. Appl. Catal. A Gen..

[42] Lv B., Feng X., Xi X., Feng X., Yuan Z., Yang Y., Zhang F. (2021). Noble-metal-free Cd_0.3_Zn_0.7_S-Ni(OH)_2_ for high efficiency visible light photocatalytic hydrogen production. J. Colloid Interface Sci..

[43] Jiang X., Liu Q., Cheng C., Xing F.S., Chen C., Huang C. (2021). In situ photodeposition of metalloid Ni_2_P co-catalyst on Mn_0.5_Cd_0.5_S for enhanced photocatalytic H_2_ evolution with visible light. Int. J. Hydrog. Energy.

[44] Ma D., Shi J.W., Sun L., Sun Y., Mao S., Pu Z., He C., Zhang Y., He D., Wang H. (2022). Knack behind the high performance CdS/ZnS-NiS nanocomposites: Optimizing synergistic effect between cocatalyst and heterostructure for boosting hydrogen evolution. Chem. Eng. J..

[45] Zhang J., Qiao S.Z., Qi L., Yu J. (2013). Fabrication of NiS modified CdS nanorod p–n junction photocatalysts with enhanced visible-light photocatalytic H_2_-production activity. Phys. Chem. Chem. Phys..

[46] Mullin J.W. (2001). Recrystallization. Crystallization.

[47] Liu M., Wang L., Lu G., Yao X., Guo L. (2011). Twins in Cd_1–x_Zn_x_S solid solution: Highly efficient photocatalyst for hydrogen generation from water. Energy Environ. Sci..

[48] Fodor L., Solymosi B., Horváth O. (2018). Investigation of Hydrogen Production from Alkaline Sulfide Solution with Nanosized CdS/ZnS-PdS Photocatalyst of Various Compositions. J. Nanosci. Nanotechnol..

[49] Sakizadeh J., Cline J.P., Wolfe E., Thorpe R., Snyder M.A., Kiely C.J., McIntosh S. (2023). Green synthesis of CdS/Ni_x_S_y_ nanoparticles as a route towards sustainable and scalable photocatalysts. Green Chem..

[50] Mersel M.-A., Fodor L., Pekker P., Makó É., Horváth O. (2022). Effects of Preparation Conditions on the Efficiency of Visible-Light-Driven Hydrogen Generation Based on Ni(II)-Modified Cd_0.25_Zn_0.75_S Photocatalysts. Molecules.

[51] Feng L., Zhang L., Chen X., Zhang C., Mao G., Wang H. (2022). A visible light-driven photoelectrochemical sensor for mercury (II) with “turn-on” signal output through in-situ formation of double type-II heterostructure using CdS nanowires and ZnS quantum dots. Chem. Eng. J..

[52] Moulder J.F., Stickle W.F., Sobol W.M., Bomben K.D. (1992). Handbook of X-ray Photoelectron Spectroscopy.

[53] Gaarenstroom S.W., Winograd N. (1977). Initial and final state effects in the ESCA spectra of cadmium and silver oxides. J. Chem. Phys..

[54] Li Y., Gao D., Peng S., Lu G., Li S. (2011). Photocatalytic hydrogen evolution over Pt/Cd_0.5_Zn_0.5_S from saltwater using glucose as electron donor: An investigation of the influence of electrolyte NaCl. Int. J. Hydrog. Energy.

[55] Biesinger M.C., Payne B.P., Lau L.W.M., Gerson A., Smart R.S.C. (2009). X-ray photoelectron spectroscopic chemical state quantification of mixed nickel metal, oxide and hydroxide systems. Surf. Interface Anal..

[56] Weidler N., Schuch J., Knaus F., Stenner P., Hoch S., Maljusch A., Schäfer R., Kaiser B., Jaegermann W. (2017). X-ray Photoelectron Spectroscopic Investigation of Plasma-Enhanced Chemical Vapor Deposited NiO_x_, NiO_x_(OH)_y_, and CoNiO_x_(OH)_y_: Influence of the Chemical Composition on the Catalytic Activity for the Ox. J. Phys. Chem. C.

[57] Ma N., Xu J., Bian Z., Yang Y., Zhang L., Wang H. (2020). BiVO_4_ plate with Fe and Ni oxyhydroxide cocatalysts for the photodegradation of sulfadimethoxine antibiotics under visible-light irradiation. Chem. Eng. J..

[58] Anantharaj S., Karthik P.E., Kundu S. (2017). Petal-like hierarchical array of ultrathin Ni(OH)_2_ nanosheets decorated with Ni(OH)_2_ nanoburls: A highly efficient OER electrocatalyst. Catal. Sci. Technol..

[59] Linkous C.A., Muradov N.Z., Ramser S.N. (1995). Consideration of reactor design for solar hydrogen production from hydrogen sulfide using semiconductor particulates. Int. J. Hydrog. Energy.

[60] Kubelka P. (1954). New Contributions to the Optics of Intensely Light-Scattering Materials Part II: Nonhomogeneous Layers. J. Opt. Soc. Am..

[61] Tauc J., Grigorovici R., Vancu A. (1966). Optical Properties and Electronic Structure of Amorphous Germanium. Phys. Status Solidi..

