# Supplementary material for: Photocatalytic H2 Production by Visible Light on Cd0.5Zn0.5S Photocatalysts Modified with Ni(OH)2 by Impregnation Method
Source: Int J Mol Sci. 2023 Jun 6;24(12):9802. doi: 10.3390/ijms24129802 (PMC10298706; doi:10.3390/ijms24129802)
Supplement: Supplementary file 1 [file ijms-24-09802-s001.zip › ijms-2408312-supplementary/Supplementary Materials.pdf]

Supplementary Materials

for

Photocatalytic H_2 production by visible light on $Cd_{0.5}Zn_{0.5}S$ photocatalysts modified with $Ni(OH)_2$ by impregnation method

Bence Páll [1], Maali-Amel Mersel[1], Péter Pekker[2], Éva Makó[3], Veronika Vágvölgyi[4], Miklós Németh[5], József Sándor Pap[5], Lajos Fodor[1] and Ottó Horváth[1,*]

[1] Research Group of Environmental and Inorganic Photochemistry, Center for Natural Sciences, Faculty of Engineering, University of Pannonia, P.O.B. 1158, Veszprém H-8210, Hungary; pallbence2001@gmail.com (B.P.); sam003miloo@gmail.com (M-A.M.); fodor.lajos@mk.uni-pannon.hu (L.F.)

[2] Environmental Mineralogy Research Group, Research Institute of Biomolecular and Chemical Engineering, University of Pannonia, H-8210 Veszprem, POB. 1158, Hungary; pekkerpeter@gmail.com (P.P.)

[3] Department of Materials Engineering, Research Center for Engineering Sciences, University of Pannonia, H-8210 Veszprem, POB. 1158, Hungary; kristofne.mako.eva@mk.uni-pannon.hu (É.M.)

[4] Research Group of Analytical Chemistry, Center for Natural Sciences, Faculty of Engineering, University of Pannonia, H-8210 Veszprem, POB. 1158, Hungary; vagvolgyi.veronika@mk.uni-pannon.hu (V.V.)

[5] Surface Chemistry and Catalysis Department, Centre for Energy Research, Hungarian Academy of Sciences, H-1121, Konkoly-Thege Street 29-33, Budapest, Hungary; nemeth.miklos@ek-cer.hu (M.N.), pap.jozsef@ek-cer.hu (J.S.P.)

* Correspondence: horvath.otto@mk.uni-pannon.hu (O.H.); Tel+36-88-624-000 / 6049 ext.

Content

Figures S1-S3 2

Figures S4, S5 3

Figures S6, S7 4

Figures S8-S10 5

Figure S11 and Table S1 6

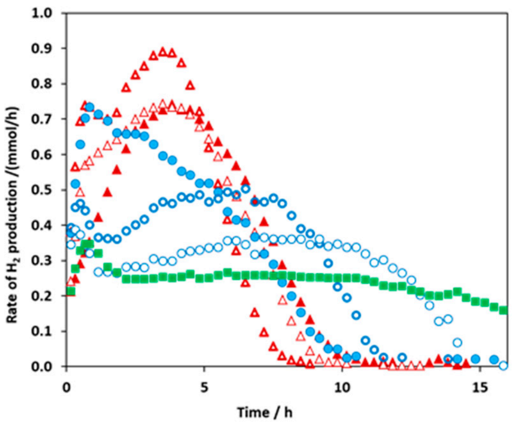

Figure S1. The RHPs over illumination time. The red, blue, and green colors represent the catalysts CZS-10-I, CZS-10-S, and CZS-10-B, respectively. The filled symbols represent the 1st illumination, the bold and thin open symbols represent the 2nd and 3rd illuminations, respectively.

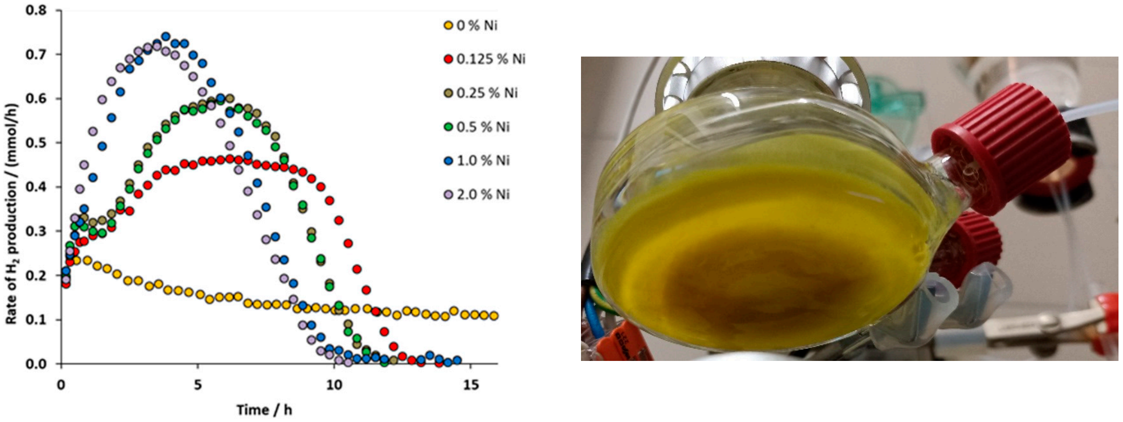

(**a**) (**b**)

Figure S2. **(a)** The RHPs over illumination time obtained for catalysts modified by impregnation method with different amounts of Ni(II) (CZS-xxNi-I). **(b)** A photo of the suspension containing the CZS-10Ni-I catalyst taken immediately after the illumination was switched off at the end of the reaction (about 15 hours after the start of the reaction).

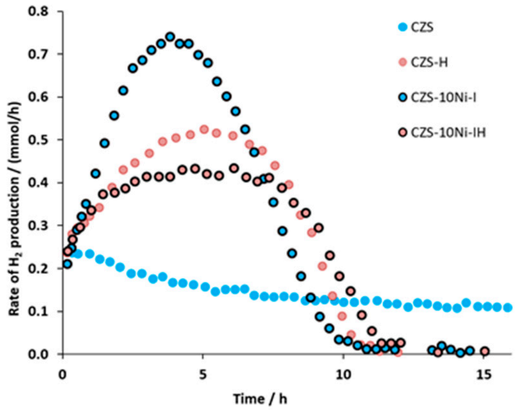

Figure S3. The RHPs over illumination time of hydrothermally treated (red) and non-treated (blue) catalysts unmodified (non-bordered sumbols) and modified with 1% of Ni(II) by impregnation (bordered symbols).

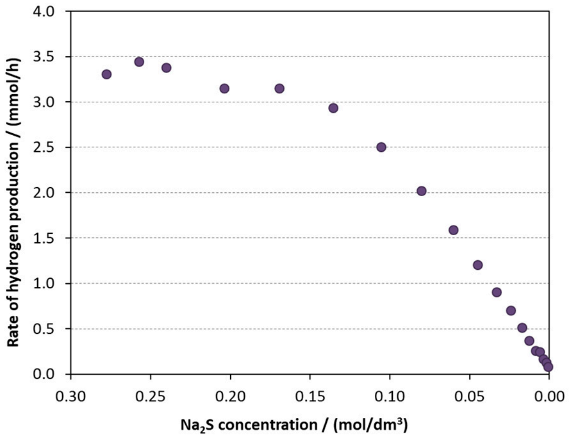

Figure S4. The RHP during the determination of quantum yield of $Cd_{0.5}Zn_{0.5}S$-10Ni-I catalyst. The light source was a 415-nm LED, the initial Na_2S and Na_2SO_3 concentrations were 0.29 M and 0.38 M, respectively. The amount of catalyst was 20 mg in 30 mL of total volume of illuminated suspension.

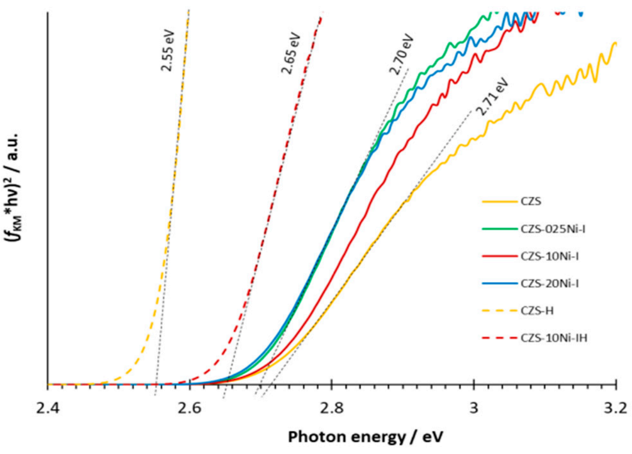

Figure S5. Tauc plots of catalysts prepared by impregnation method with different amounts of Ni(II). The dashes curves correspond to hydrothermally treated catalysts (dashed orange: CZS, dashed red: CZS-10Ni-IH).

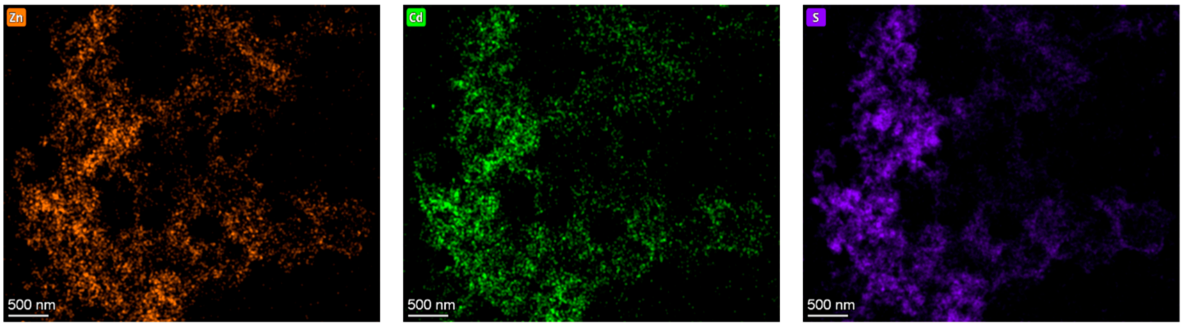

Element	Atomic fraction (%)	Atomic error (%)
S K	50.03	7.35
Ni K	41.43	5.25
Zn K	6.44	0.90
Cd L	2.10	0.26

S / (Ni + Zn + Cd) ratio = 1.001

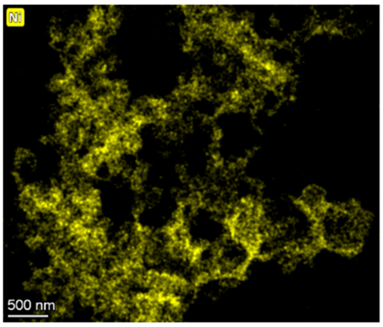

Figure S6. STEM elemental maps and the atomic fractions of a selected Ni-rich CZS-10Ni-S particle. The data show that the CZS-10Ni-S product contains extremely Ni-rich particles, mainly consisting of NiS.

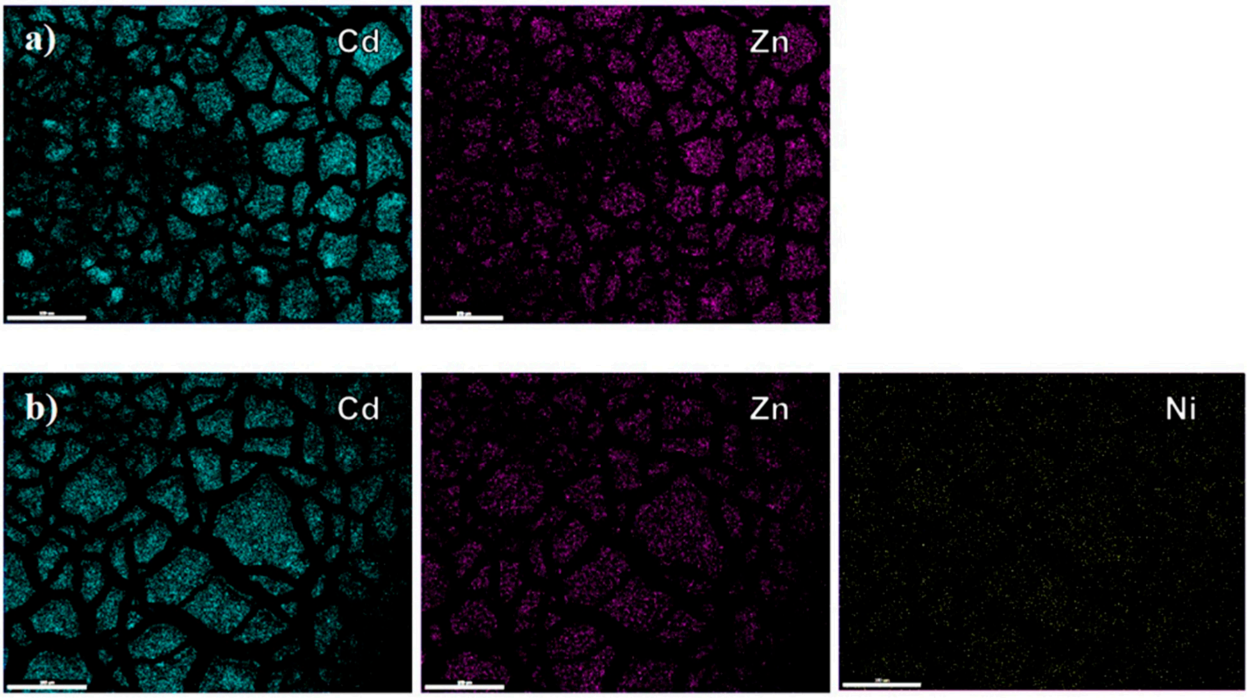

Figure S7. SEM-EDS elemental maps of CZS (a) and CZS-10Ni-I (b) composites.

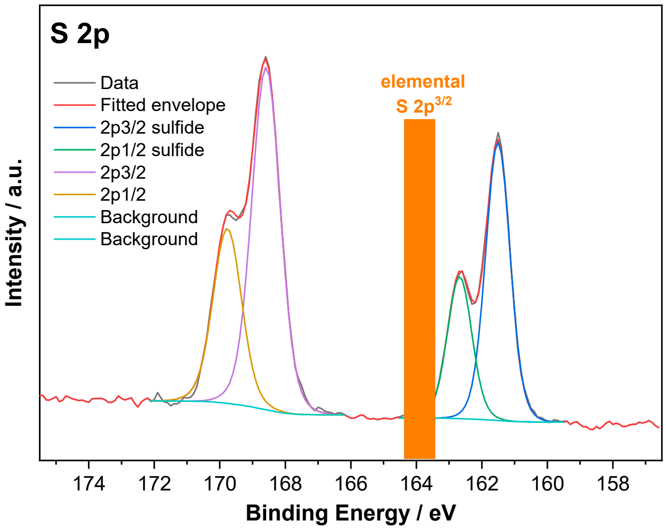

Figure S8. A representative S 2p XP spectrum of CZS-10Ni-I catalyst after its irradiation. It shows that no sulfur was deposited on the surface.

(**a**) (**b**)

Figure S9. The Cd 3d (a) and the Zn 2p (b) XP spectrum of CZS-10Ni-I catalyst after its irradiation.

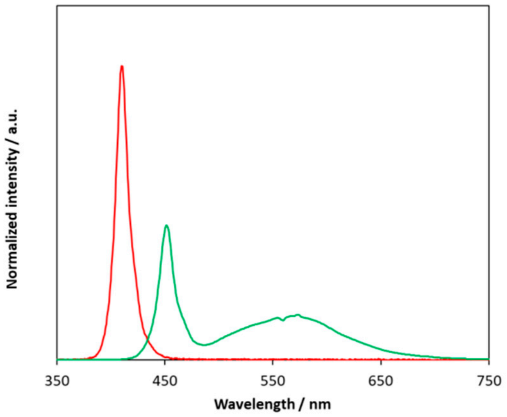

Figure S10. Normalized emission spectra of light sources applied. The green spectrum belongs to 7W Optinica LED, and the red curve represents the 415-nm LED.

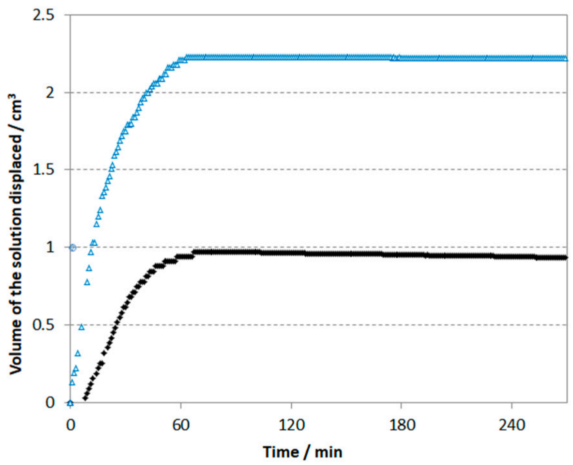

Figure S11. The volume of solution displaced from the buffer vessel during illumination of 30 ml of sacrificial solution containing 0.145 M Na_2S, 0.19 M Na_2SO_3. Black symbols represent the system without a catalyst, and blue symbols represent the system containing 20 mg of pure ZnS.

During the first 60–70 minutes, a small amount of solution was displaced due to the thermal dilatation of the reaction mixture. Since the absorbance of the system containing ZnS is higher, its equilibrium temperature is higher, which causes a higher dilatation. After reaching thermal equilibrium, no more solution was displaced. The very small decrease in the mass of the displaced solution is due to evaporation (about 0.01 g/h). Evaporation causes an error of about 0.4 μmol/h in the RHP, but this value is much smaller than the indicated total error of 25 μmol/h.

Table S1. Time data for the calculation of RHP values.

Catalyst	t_{20} / h[1]	t_{50} / h[1]	RHP[2] /(cm^3/h)	RHP /(μmol/h)
CZS-10Ni-I	2.06	3.84	16.87	697.2
CZS-10Ni-I2	1.32	2.91	18.90	780.9
CZS-10Ni-I3	1.64	3.40	17.03	703.7
CZS-10Ni-S	1.35	3.27	15.62	645.4
CZS-10Ni-S2	2.11	4.87	10.86	448.8
CZS-10Ni-S3	2.79	6.59	7.91	326.8
CZS-10Ni-B	3.00	7.79	6.26	258.6
CZS	4.13	13.52	3.19	131.9
CZS-0125Ni-I	2.79	5.60	10.68	441.2
CZS-025Ni-I	2.57	4.89	12.95	534.9
CZS-05Ni-I	2.64	4.97	12.86	531.3
CZS-20Ni-I	1.64	3.40	17.03	703.7
CZS-H	2.47	4.98	11.92	492.6
CZS-10Ni-IH	2.48	5.45	10.11	417.8

[1] t_{20} and t_{50} are the times required to evolve 20 cm^3 and 50 cm^3 of hydrogen, respectively.

[2] $RHP = 30/(t_{50} - t_{20})$.
